# Fluid-Dynamic Optimal Design of Helical Vascular Graft for Stenotic Disturbed Flow

**DOI:** 10.1371/journal.pone.0111047

**Published:** 2014-10-31

**Authors:** Hojin Ha, Dongha Hwang, Woo-Rak Choi, Jehyun Baek, Sang Joon Lee

**Affiliations:** 1 Department of Mechanical Engineering, Pohang University of Science and Technology (POSTECH), Pohang, Gyeongbuk, Republic of Korea; 2 Center for Biofluid and Biomimic Research, Pohang University of Science and Technology (POSTECH), Pohang, Gyeongbuk, Republic of Korea; University of California San Diego, United States of America

## Abstract

Although a helical configuration of a prosthetic vascular graft appears to be clinically beneficial in suppressing thrombosis and intimal hyperplasia, an optimization of a helical design has yet to be achieved because of the lack of a detailed understanding on hemodynamic features in helical grafts and their fluid dynamic influences. In the present study, the swirling flow in a helical graft was hypothesized to have beneficial influences on a disturbed flow structure such as stenotic flow. The characteristics of swirling flows generated by helical tubes with various helical pitches and curvatures were investigated to prove the hypothesis. The fluid dynamic influences of these helical tubes on stenotic flow were quantitatively analysed by using a particle image velocimetry technique. Results showed that the swirling intensity and helicity of the swirling flow have a linear relation with a modified Germano number (Gn*) of the helical pipe. In addition, the swirling flow generated a beneficial flow structure at the stenosis by reducing the size of the recirculation flow under steady and pulsatile flow conditions. Therefore, the beneficial effects of a helical graft on the flow field can be estimated by using the magnitude of Gn*. Finally, an optimized helical design with a maximum Gn* was suggested for the future design of a vascular graft.

## Introduction

Circulatory vascular diseases (CVDs) are the leading cause of death in developed countries. According to the American Heart Association, more than 71 million Americans suffer from CVD. In 2003, CVD was estimated to be the underlying cause of death for 37.3% of all 2.4 million deaths, or 1 of every 2.7 deaths, in the United States [1].

The hemodynamic characteristics of blood flow in blood vessels are closely related to the development of CVDs. Mechanical factors such as blood pressure and wall shear stress (WSS) exert tensional and shearing forces on the endothelial cells (ECs) that line the blood vessel wall; these ECs adapt their morphology and proliferation in response to the surrounding hemodynamic stimuli [2]. Therefore, various pathological hemodynamic conditions, such as low WSS, high oscillatory shear index (OSI), and high temporal and spatial gradient of WSS distribution, cause abnormal morphological and functional changes in the EC layer that contribute to elevated wall permeability and possible vascular lesions [3], [4].

Stenosis is a medical terminology referring to the abnormal narrowing of a blood vessel by pathological causes, such as atherosclerosis and intimal hyperplasia (IH). It is one of CVD that is highly influenced by local hemodynamics [5]. Once stenosis is developed by an atherosclerotic plaque in a blood vessel, blood flow is significantly disturbed because of the local narrowing of the vessel lumen. This disturbance is characterized by high shear stress at the stenosis apex, flow separation and recirculation, vortex shedding and turbulent transition at a downstream region of the stenosis. These hemodynamic environments influence atherogenic EC activation, platelet activation and thrombosis formation in the stenosis [6]. Therefore, a comprehensive understanding of the hemodynamic characteristics of a stenosis is important in identifying effective diagnosis and clinical treatments.

Since Stonbridge *et al.* [7], [8] reported the existence of swirling blood flow in a human femoral artery, several follow-up studies observed swirling flow in various arterial vasculatures, such as the aortic arch and the arterial branch [4], [9]–[11]. The swirling flow is suspected to be caused by the three-dimensional (3D) arterial geometry, non-planar curvature and branching of arteries [8], [9], [12].

The swirling flow is considered to have various advantages in the fluid transport phenomena. Stonebridge and Brophy [12] reported that a spiral configuration of blood flow may have a stabilizing effect on the turbulence caused by a stenosis. Morbiducci *et al.* [10], [11] speculated that coherent swirling blood flow might prevent the excessive dissipation of energy by limiting flow instability in arteries. Coppola and Caro [13] reported that arterial three-dimensionality increases both WSS and oxygen transport. By employing the beneficial effects of swirling flow, Caro *et al.* [14] developed a small-amplitude helical graft that generates physiological swirling flow and showed that the helical graft induces little thrombosis and IH, whereas conventional grafts have frequent failures because of the stenosis caused by IH. Cookson *et al.* [15] also numerically investigated in-plane mixing of the flow in the small amplitude helical tubes.

Since the surgical implantation of the end-to-side vascular graft creates artificial bifurcation flow, it modifies an inborn hemodynamic environment in the blood vessel, and pathological hemodynamic features, such as recirculation/stagnation flows, can be formed within the blood vessels. Ojha et al. [16], [17] reported that IH is frequently observed in regions of recirculation/stagnation flows where low wall shear stress (WSS) with long residence time and high spatial and temporal WSS gradient are formed. In addition, high oscillatory WSS is also suspected to increase the development of IH [18]. Therefore, the beneficial effects of the swirling flow have been investigated for improving the disturbed flow field by the introduction of the vascular graft. Sherwin et al. [19] and Papahariliaou et al. [20] investigated the influence of out-of-plane geometry on the flow field within a distal end-to-side anastomosis. They found that the swirling flow induced by the non-planar graft reduces oscillatory shear stress on the recipient vessel, and reported that this may have beneficial roles on suppressing the development of IH.

Although blood flow in a helical conduit appears to be clinically beneficial, hemodynamic features (e.g. velocity and vorticity distribution, swirling intensity and helicity) of the blood flow in the helical conduit under various pathological conditions have not been fully revealed yet. Especially, blood flow in a vascular graft frequently encounters local constriction of the blood vessel due to the development of IH, thrombosis formation or vasoconstriction [4], [21], [22]. Therefore, we aimed to investigate fluid-dynamic characteristics of the flow in the helical conduit under local constriction. Specifically, the present study investigates fluid-dynamic influence of the swirling flow characteristics generated by helical tubes (which mimic helical vascular grafts) with various helical pitches and curvatures on suppressing pathological flow fields behind the local constriction. Finally, the helical design of the conduit, which produces the optimum fluid-dynamic characteristics behind the local constriction, is obtained.

## Materials and Methods

### 2.1 Helical geometry and parameters

**Figure 1:**
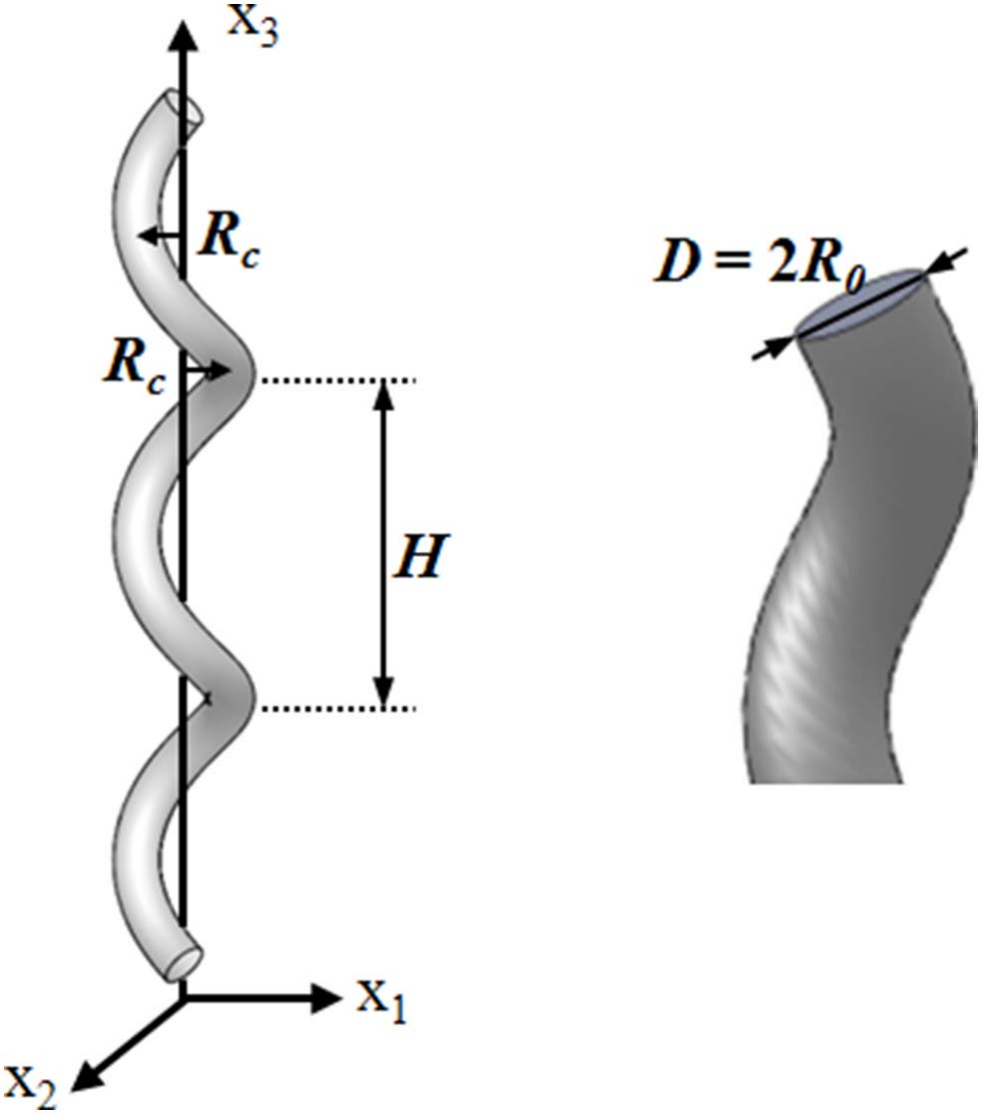
Geometrical parameters of a helical graft.

**Figure 2:**
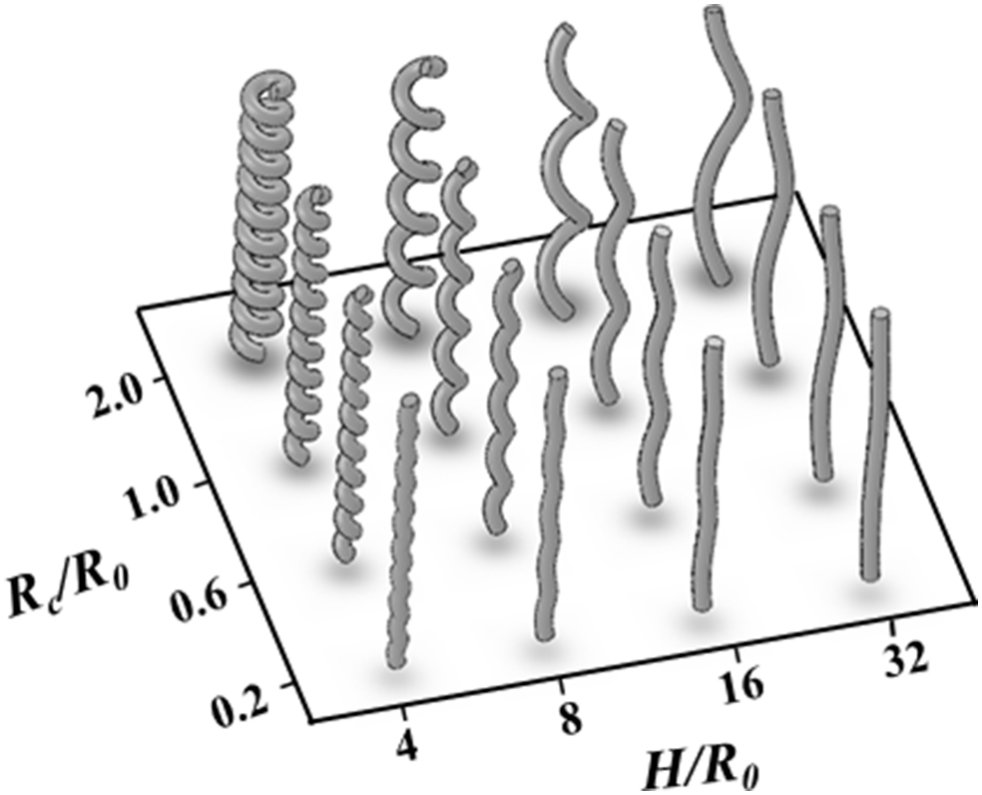
Helical tubes with different helical pitches (*H/R_0_*) and radii of curvatures (*R_c_/R_0_)*.

Figure 1 shows the geometrical parameters of the helical tube used in the study. The helical tube is located in the Cartesian coordinate system **x** (x_1_, x_2_, x_3_). *R_c_* and *H* are the radii of the curvature and pitch, respectively, of the helical tube. *D* and *R_0_* are the diameter and radius, respectively, of the tube. To investigate the effects of the helical pitch and curvature, 16 helical tubes with four helical pitches (*H/R_0_* = 4, 8, 16 and 32) and four radii of curvatures (*R_c_/R_0_* = 0.2, 0.6, 1.0 and 2.0) were fabricated with acrylonitrile butadiene styrene thermoplastic by using a 3D printer (Fortus 400mc, Stratasys), as shown in Figure 2. The diameter (*D*) and height of the helical tubes are 10 and 200 mm, respectively.

A helical geometry can be described by two curve parameters: curvature (*λ*) and torsion (*η*) of a helix. These parameters are expressed as
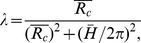
(1)

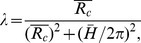
(2)where 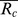 and 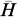 indicate the dimensionless radii of the curvature and pitch, respectively, of the helical tube; 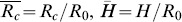.

According to Germano [23] and Liu and Masliya [24], the Germano number (*Gn*) is defined as
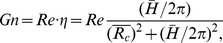
(3)which is a direct measure of the ratio of the twisting forces to the viscous forces in the helical pipe flow. In other words, *Gn* is a measure of the torsion effect on secondary flow. As the helical parameters (e.g. λ, *η* an*d Gn)* are derived only for *R_c_> R_0_*, they require a slight modification to be used for *R_c_ <R_0_*. At *R_c_* <*R_0_*, the centreline of the helical pipe is too close to its coiling axis so that a sub-portion of the pipe at the inner curvature becomes merged. As a result, the helical pipe consists of a non-helical inner section and a helical-coiling outer groove (Figure S1). As *R_c_* decreases, the helical-coiling portion of the helical pipe is lessened, which results in the reduction of the torsion effect on the helical pipe flow. In the present study, a correction factor (*β*) and an effective radius of curvature (R_c_*) were introduced as follows to consider the reduced torsion effect and to extend the use of *Gn* at *R_c_<R_0_*:
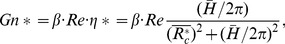
(4)for *R_c_*<*R_0_*,



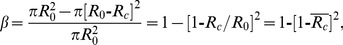
(5)

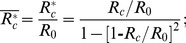
(6)


for *R_c_*≥*R_0_*,



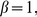
(7)

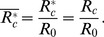
(8)Here, η* is the torsion calculated with the effective radius of curvature (R_c_*). The derivations of *β* and R_c_* are described in Figure S1.

Table 1 summarizes the representative helical parameters of 16 helical tubes at *Re* = 814. Given the various combinations of *R_c_ and H*, a wide range of η* (0.170≤ η* ≤0.892) can obtained to investigate the effects of torsion variation on secondary flow.

**Table 1:** Representative experimental parameters of helical tubes at *Re* = 814.

*R_c_ (m)*	*H(m)*	*R_c_/R_0_*	*H/R_0_*	*R_c_'/R_0_*	*β*	η*	λ*	Gn*
0.001	0.02	0.2	4	0.556	0.36	0.892	0.778	261.29
0.001	0.04	0.2	8	0.556	0.36	0.660	0.288	193.28
0.001	0.08	0.2	16	0.556	0.36	0.375	0.082	109.80
0.001	0.16	0.2	32	0.556	0.36	0.194	0.021	56.83
0.003	0.02	0.6	4	0.714	0.84	0.695	0.780	475.51
0.003	0.04	0.6	8	0.714	0.84	0.597	0.335	408.36
0.003	0.08	0.6	16	0.714	0.84	0.364	0.102	248.82
0.003	0.16	0.6	32	0.714	0.84	0.192	0.027	131.60
0.005	0.02	1	4	1.000	1	0.453	0.711	368.76
0.005	0.04	1	8	1.000	1	0.486	0.381	395.41
0.005	0.08	1	16	1.000	1	0.340	0.133	276.95
0.005	0.16	1	32	1.000	1	0.189	0.037	153.90
0.01	0.02	2	4	2.000	1	0.145	0.454	117.63
0.01	0.04	2	8	2.000	1	0.227	0.356	184.38
0.01	0.08	2	16	2.000	1	0.243	0.191	197.70
0.01	0.16	2	32	2.000	1	0.170	0.067	138.47

### 2.2 Swirling intensity of the swirling flow

Rocklage-Marliani *et al.* [25] employed swirl number *S* to characterize the swirling intensity of the secondary flow developed by a helical tube. Swirl number *S* of the flow in the pipe is defined as the ratio of the angular momentum flux to the axial momentum flux as follow:
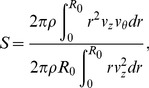
(9)where *R_0_* is the radius of the tube, and *v_z_ and v_θ_* are the axial and tangential velocity components based on the local cylindrical coordinate with respect to the cross-sectional surface of the pipe. In addition, θ is the azimuthal angle on the cross-sectional surface.

Helicity (*H*) has been used as an alternative parameter for characterizing swirling flow [10], [11], [26]. In the present study, the helicity quantity of the swirling flow at an outlet surface of a helical tube is defined as
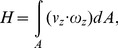
(10)where *v_z_* and *ω_z_* indicate the axial velocity and vorticity normal to the outlet surface, respectively.

Since the swirl number is defined as the vector product of radius and rotational momentum, it evaluates the distance-weighted value based on the distance from the tube center. On the other hand, helicity measures the rotational motion of fluid elements because it estimates the scalar product of velocity and vorticity of each fluid element. As a result, a rotational flow far from the center has large influence on swirl number than that near the tube center, whereas, helicity is independent of the radial distance from the tube center. Therefore, both of the swirl number (S) and helicity (H) are employed in this study to characterize the swirling flows created by helical grafts.

### 2.3 Flow circuit system

**Figure 3:**
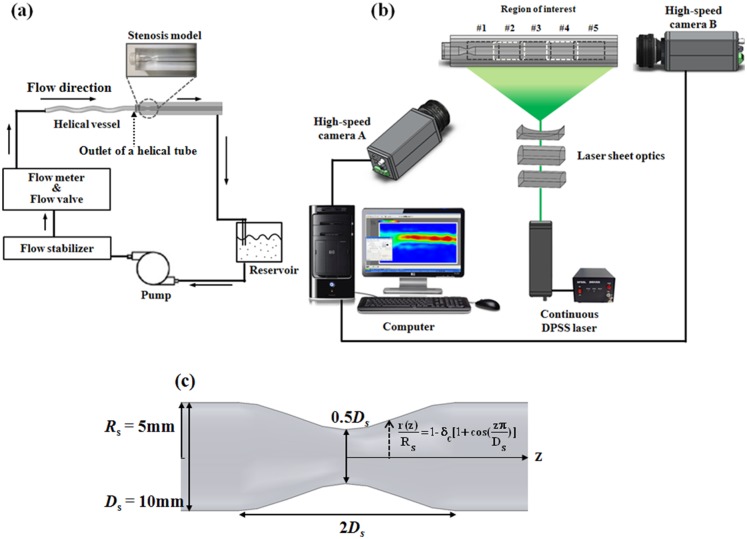
Schematic diagrams of the experimental set-up. (a) Flow circuit system, (b) PIV velocity field measurement system, (c) schematic of the stenosis model.

Figure 3a shows a schematic diagram of the flow circulating system used in this study. A total of 3 L working fluid was prepared in an acryl reservoir. A 15 W centrifugal pump circulated the working fluid through the circuit at a constant flow rate. A 0.5 L air container was installed as a fluidic low-pass filter using a three-way connector to stabilize possible fluctuations in flow rate [27], [28]. To measure the flow rate, a variable area-type flow meter (Visi-Float, Dwyer Instruments, Inc., Michigan, USA) was installed after proper re-calibration with the working fluid. The flow rate was controlled by the internal fluid valve of the flow meter.

A swirling flow was generated as the working fluid passed through the helical tube. This swirling flow then entered the stenosis model. The stenosis model depicted in Figure 3c was manufactured with an acrylic material with the following cosine-form formula [29]:
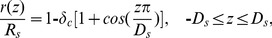
(11)where *R_s_* and *D_s_* are the radius and the diameter, respectively, of the straight part of the stenosis model; *r* and *z* represent the radial coordinate and the axial coordinate, respectively; parameter *δ*_c_ denotes the percentage of vessel constriction. In the stenosis model of this study, *δ*_c_ = 0.25, which is indicative of a 50% and 75% reduction in the diameter and the cross-sectional area, respectively. The sinusoidal shape of stenosis with 50% reduction in diameter provides a smooth constriction whose geometry is a reasonable representation of an arterial stenosis. Therefore, it has been frequently used as a canonical stenosis model for various experimental studies [29]–[34]. The present stenosis model is also similar with the previous model. The total length of the stenosis model is 200 mm (20D_s_), whereas *D_s_* = 10 mm. The length of the stenosis region and downstream of the stenosis model are 2D_s_ and 15D_s_, respectively. The length of the upstream of the stenosis model is set to 3D_s_ to obtain enough distance for connecting the helical graft and the stenosis model.

The refractive index of the working fluid must match the experimental acrylic model to achieve proper optical access and accurate velocity measurements. According to Deutsch *et al.* [35], a blood-analog working fluid is composed of 79% saturated aqueous NaI, 20% pure glycerol and 1% water (by volume). In the present study, a kinematic viscosity of the working fluid was experimentally measured to 2.8×10^−6^ m^2^/s by using a rotational viscometer (DV-II+Pro, Brookfield Engineering Laboratories, Inc., USA), which lies within the range of human blood viscosity (2.8∼3.8×10^−6^ m^2^/s) [36]. The refractive index was measured to 1.491±0.001 at 25°C using an Abbe refractometer (Atago, Japan). This setting enabled optical access to the flow inside the acrylic model for planar velocity measurements. The working fluid was seeded with silver-coated hollow glass spheres (Conduct-O-Fill SH400S20 silver hollow, Potters Industries, Inc.) with a mean diameter of *d*_p_ = 13 µm and circulated through the flow loop. The seeding density of the particles was ∼0.01% by weight. The effective size of the particle on the image was ∼5 pixels, thus resulting in approximately 10 particles per interrogation window (16×16 pixels). All experiments were performed at a controlled room temperature of 25°C.

### 2.4 Particle image velocimetry (PIV) measurements

Figure 3b shows a schematic diagram of the PIV velocity field measurement system. To illuminate the measurement plane, a thin laser sheet measuring 0.5 mm thick was generated using a 1 W continuous diode-pumped solid state laser (Shanghai Dream Lasers Technology Co., Ltd., China). A high-speed camera with a 1 K×1 K pixel resolution (Fastcam SA1.1, Photron, USA) was used to capture the flow images for velocity field estimation. As described by Ha and Lee [33], two cameras at different positions were used to measure flow velocities in the horizontal and cross-sectional planes. A high-speed camera captured consecutively flow images at 2000 frames to 5000 frames per second depending on flow rates. Subsequently, 1000 and 5000 consecutive images were obtained for velocity estimation in the cross-sectional plane and the horizontal plane, respectively. Thus, 999 and 4999 pairs of instantaneous vector fields were obtained and statistically averaged to the mean velocity fields. To determine the whole velocity fields along the post-stenosis region (∼15D), the post-stenosis region was divided into five different regions of interest (ROIs), and the velocity field in each ROI was measured separately. The whole velocity fields in the five ROIs were later combined together.

The axial velocity distributions at the outlet region of the helical tubes were measured to analyse the skewness of the axial flow caused by the torsion effect of the tube. By employing a two-dimensional (2D)-scanning PIV measurement technique [37], the axial velocity distributions in the cross-section of the channel were divided into 40 parallel measurement planes and measured separately with a scanning step interval of 0.25 mm. The axial velocity distributions at the outlet of the helical tubes were obtained by reconstructing the axial velocities in all planes.

PIV analysis was performed using PIVlab [38] built on the MATLAB platform. A fast Fourier transform-based cross-correlation PIV algorithm was applied in the acquired flow images to extract instantaneous velocity fields. A multi-grid interrogation window scheme was adopted using 64×64, 32×32 and 16×16 pixels of interrogation windows with 50% overlapping. The distance between two adjacent velocity vectors was 8 pixels, which corresponded to 0.21 (horizontal plane) and 0.09 mm (cross-sectional plane). Further details on the PIV principle and uncertainty analysis are available in Figure S2.

### 2.5 Computation method

Commercially available computational fluid dynamics (CFD) simulation software ANSYS FLUENT 13.0 was employed to numerically simulate the flow fields inside the helical pipes. 3D Reynolds-averaged Navier–Stokes equations were solved using a cell-centred finite volume method to obtain a steady solution. The segregated pressure-based approach was used for this simulation because the flow is incompressible with a low Reynolds number. The convection and diffusion terms in the governing equations were spatially discretized using the upwind scheme and the central differencing scheme with second-order accuracy. The gradients were computed using the least-squares cell-based method, and pressure–velocity coupling was achieved using the semi-implicit method for pressure-linked algorithm to satisfy the continuity equation.

**Figure 4:**
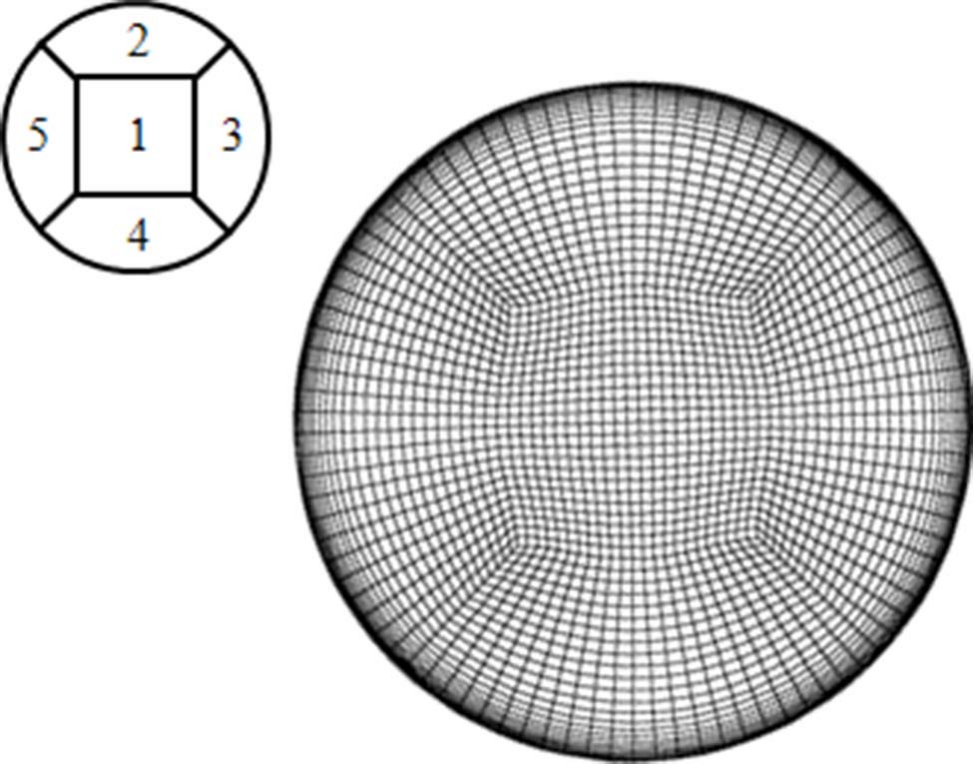
Computational grid and block arrangement.

The computational grid and block arrangement are shown in figure 4. The grid system composed of hexahedral unstructured meshes was generated by the mesh generation software ICEM CFD. The flow region was discretized from a five-block structure, that is, the O-type grid. The grid consisted of 96 points in an angular direction. Thus, 24 points made up each side of the rectangular grid section (see grid block 1 in figure 4). Approximately 790 points were generated in the axial direction. The grid resolution was set to high near the wall. The minimum grid spacing on the walls was set to 5×10^−5 ^m for the accurate estimation of viscous flow without a wall function method. The total number of grids was approximately 2.5×10^6^; the exact numbers slightly varied according to the curvature and pitch of the model. Given that solutions and their stability can be influenced by the number of grids, the grid structure was optimally selected through a grid dependency test. Grid independence was verified by increasing the number of computational grids from 5×10^5^ to 6×10^6^ and by examining the helicity of the flow at the outlet. The optimum number of grids was determined to be 2.5×10^6^ when the change in helicity change was less than 0.2%.

The following steady mass flow rates were set as the initial boundary conditions: 0.0314, 0.0234, 0.0158 and 0.0079 kg·s^−1^. These values corresponded to *Re* = 213, 410, 609 and 814. The k-ω SST turbulence model was employed for calculation because it predicts well both of laminar and turbulent flows. In addition, the k-ω SST can predict possible flow separations due to the adverse pressure gradient inside the helical graft. The turbulent kinetic energy in the final solution was ∼10^−6^ m^2^/s^2^, and the turbulent/laminar viscosity ratio was ∼10^−3^. These show that the k-ω SST model well estimates the laminar helical pipe flow with low Reynolds number. Static pressure was applied at the outlet boundary, and a no-slip condition was applied on the tube wall. The specifications of the working fluid used for the computations are the same as those in Section 2.3. The computation for each simulation took eight hours using a 3.4 GHz quad-core processor. As the flow field was considered to be in a steady state, the convergence criterion of the simulation was established when the residuals of the continuity were under 1×10^−5^.

### 2.6 WSS and OSI estimation

WSS estimation at the post-steonsis region from the velocity field data obtained by the PIV measurement is based on a method described by Jamison et al. [39] At first, to determine wall locations, the maximum intensity of each pixel for 500 flow images is obtained. The obtained image has bright interior due to tracer particles and dark exterior out of the conduit. The wall location is identified where the intensity of the image decreases to less than 5% of the maximum intensity. The magnitude of WSS is then estimated from the following definition:
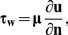
(12)where **n** is the local wall-normal vector, **u** is the local velocity component and **μ** is the dynamic viscosity of the fluid. To estimate the gradient of the velocity at the wall, at first, the wall positions were set to zero-crossing points where the flow velocity is zero due to the no-slip condition. Then, the velocity gradients are obtained by using a second-order polynomial fitting to the near-wall velocity data. In the present study, the WSS distribution is estimated only at X/D≥1 where the conduit is straight to escape the complex WSS estimation at the curved geometry.

OSI indicates the degree of WSS oscillation during a pulsating cycle, and it is estimated as follows:
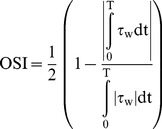
(13)here, τ_w_ indicates the WSS. The OSI ranged between 0.5 and zero. An OSI value of 0.5 indicates equal amounts of negative and positive WSS within a cycle, whereas zero OSI indicates the absence of change in the WSS sign in the pulsating cycle.

## Results

### 3.1 Validation of CFD results with PIV experimental data

**Figure 5:**
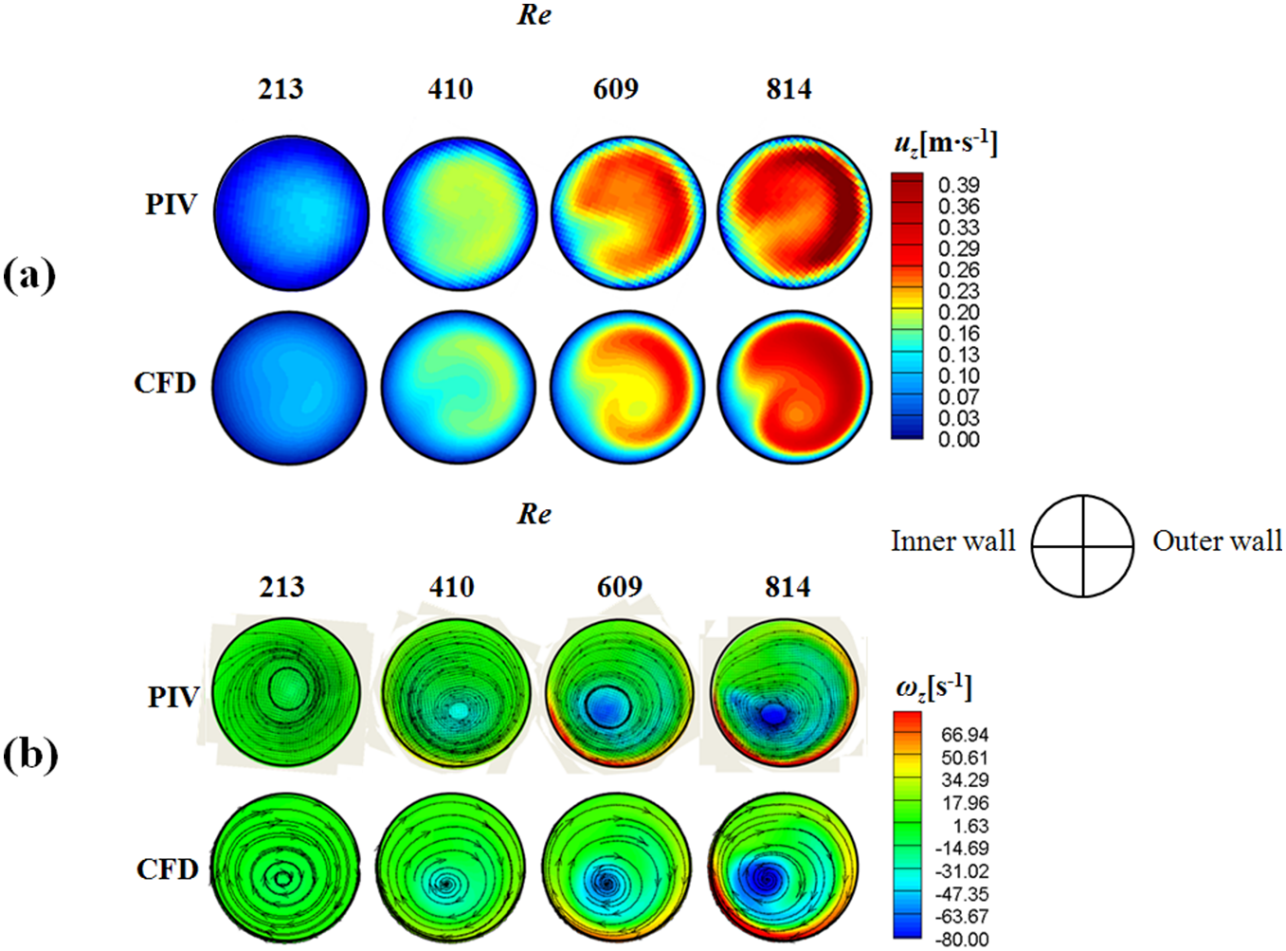
Comparison of PIV and CFD results data at the outlet of a helical tube (*H/R_0_* = 8, *R_c_/R_0_* = 1.0). (a) Axial velocity distribution and (b) normal-direction vorticity contours and corresponding streamlines.

**Figure 6:**
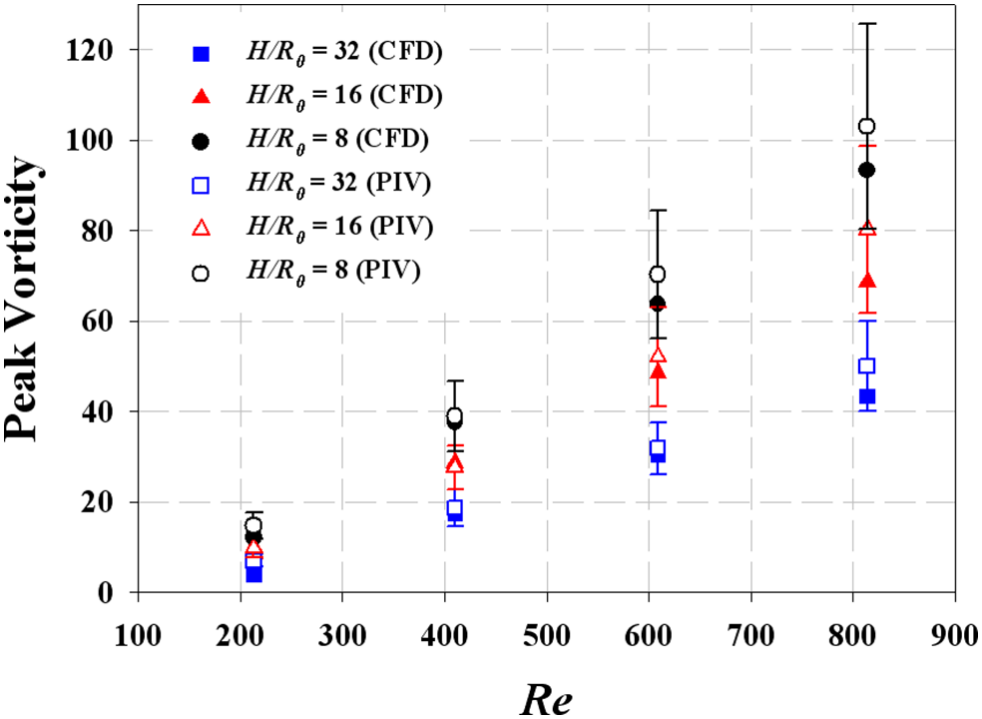
Comparison of normal-direction peak vorticity magnitude at the outlet of the helical tubes obtained from PIV and CFD. *R_c_/R_0_* are fixed to 1.0. The error bars indicate 95% confidence limits. The helical graft of *H/R_0_ = *4 was omitted for the clarity of the figure because its data overlaps with the others.

**Figure 7:**
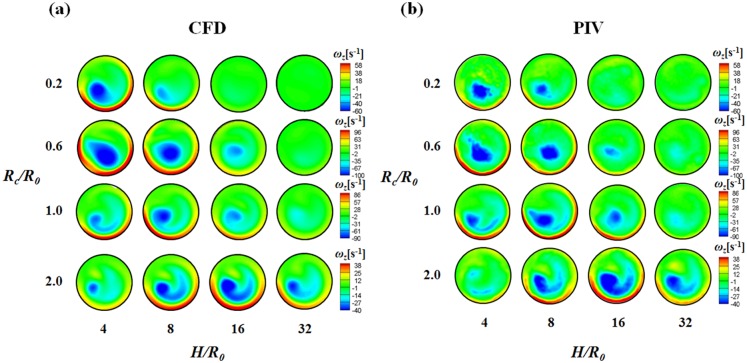
Normal-direction vorticity field contours at the outlet of the helical tubes for a range of helical curvatures and pitches. The results obtained by (a) CFD and (b) PIV are compared at *Re* = 814.

**Figure 8:**
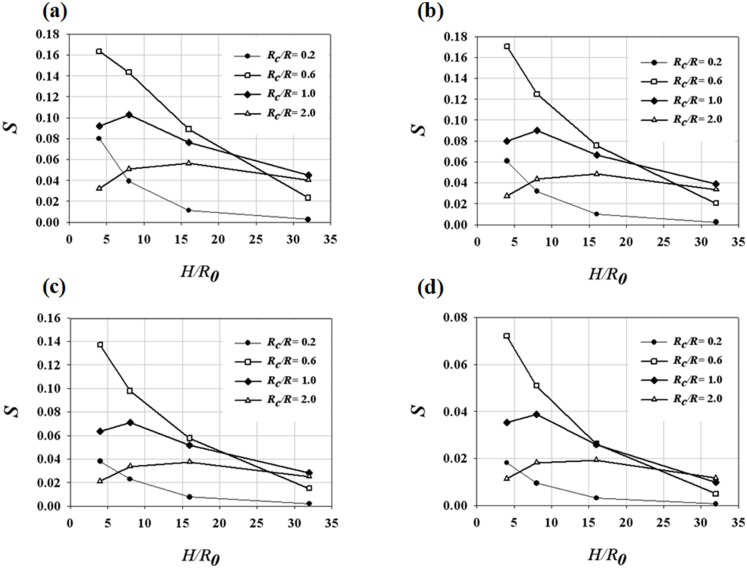
Variations of swirling intensity (*S*) for a range of helical curvatures and pitches at *Re* of (a) 814, (b) 609, (c) 410 and (d) 213.

**Figure 9:**
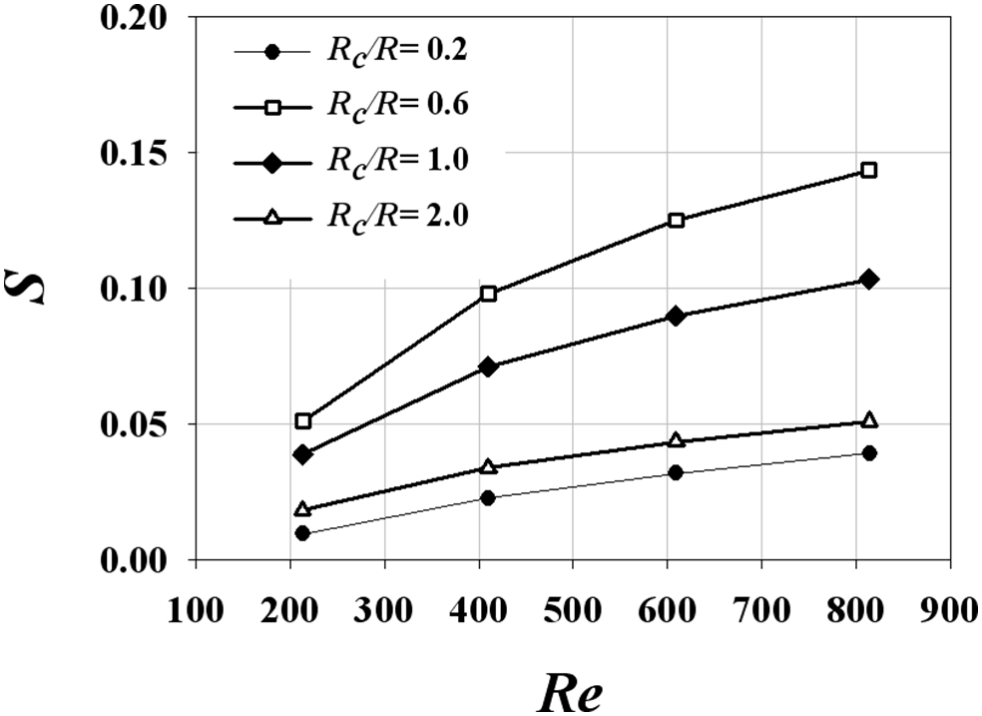
Effect of *Re* on the variation of swirling intensity (*S*) at *H/R_0_* = 8.

The CFD results were validated by comparing the axial velocity distribution as well as the normal-direction vorticity field at the outlet of the helical tube (*H*/*R_0_* = 8, *R_c_*/*R_0_* = 1) obtained by CFD with the PIV results in figure 5. The axial velocity distribution tends to be skewed toward the outer wall (figure 5a) because of the inertia of the flow at the helical tube. At *Re* = 213, the axial velocity distribution is slightly distorted from the Poiseuille flow profile. As *Re* increases, the skewing phenomenon significantly intensifies, thus causing the shift of the maximum velocity of the flow near the wall. Figure 5b shows the swirling secondary flow produced by the helical tube according to *Re*. The streamlines and vorticity fields indicate the clockwise direction of the swirling flow with a single rotating axis. As *Re* increases, the magnitude of the vorticity at the centre of the swirling flow increases, which implies strong swirling flow. The CFD results successfully demonstrate the skewing phenomena of the axial velocity distribution and the swirling characteristics of the flow. In addition, the magnitudes of the peak vorticity measured by PIV and CFD are compared quantitatively in figure 6. While the CFD data slightly underestimates the peak vorticity around 10% on average, it shows overall agreement with the PIV result. In addition, the CFD result successfully estimates the linear increase of the peak vorticity with respect to the *Re* within the PIV measurement uncertainty. Figure 7 shows the normal-direction vorticity field distribution of swirling flows at various helical curvatures and pitches. In the case of the helical tube with a small radius of curvature (*R_c_*/*R_0_* = 0.2), the magnitude of vorticity is highest at the short helical pitch (*H*/*R_0_* = 4). By contrast, the most intensive swirling flow occurs at long pitches as *R_c_*/*R_0_* increases. The maximum vorticity is observed at *H*/*R_0_* = 8 and 16 when *R_c_*/*R_0_* = 1.0 and 2.0, respectively. The swirling variations for a range of helical curvatures and pitches were quantitatively analysed using swirling intensity (*S*) (Figure 8). The swirling intensity shows the effect of the helical pitch on the swirling flow at a fixed radius of curvature. *S* is highest at the shortest helical pitch (*H*/*R_0_* = 4) for small helical curvatures (*R_c_*/*R_0_* = 0.2 and 0.6). However, the maximum point shifts to *H*/*R_0_* = 8 and 16 at *R_c_*/*R_0_* = 1.0 and 2.0, respectively. This is attributed to the variation of Gn*, which is a measure of the ratio of the twisting forces to the viscous forces, according to helical curvature. The Gn* has the maximum values at the larger helical pitches (*H*/*R_0_* = 8 and 16) when the helical curvature is increased to *R_c_*/*R_0_* = 1.0 and 2.0, respectively. The variation of the swirling intensity with *Re* is shown in figure 9. For the fixed helical pitch (*H*/*R_0_* = 8), the swirling intensity increases in proportion to *Re*, thus indicating that a high flow rate induces considerably intensive swirling flow.

### 3.2 Effect of swirling flow on stenosis flow under a steady flow condition

**Figure 10:**
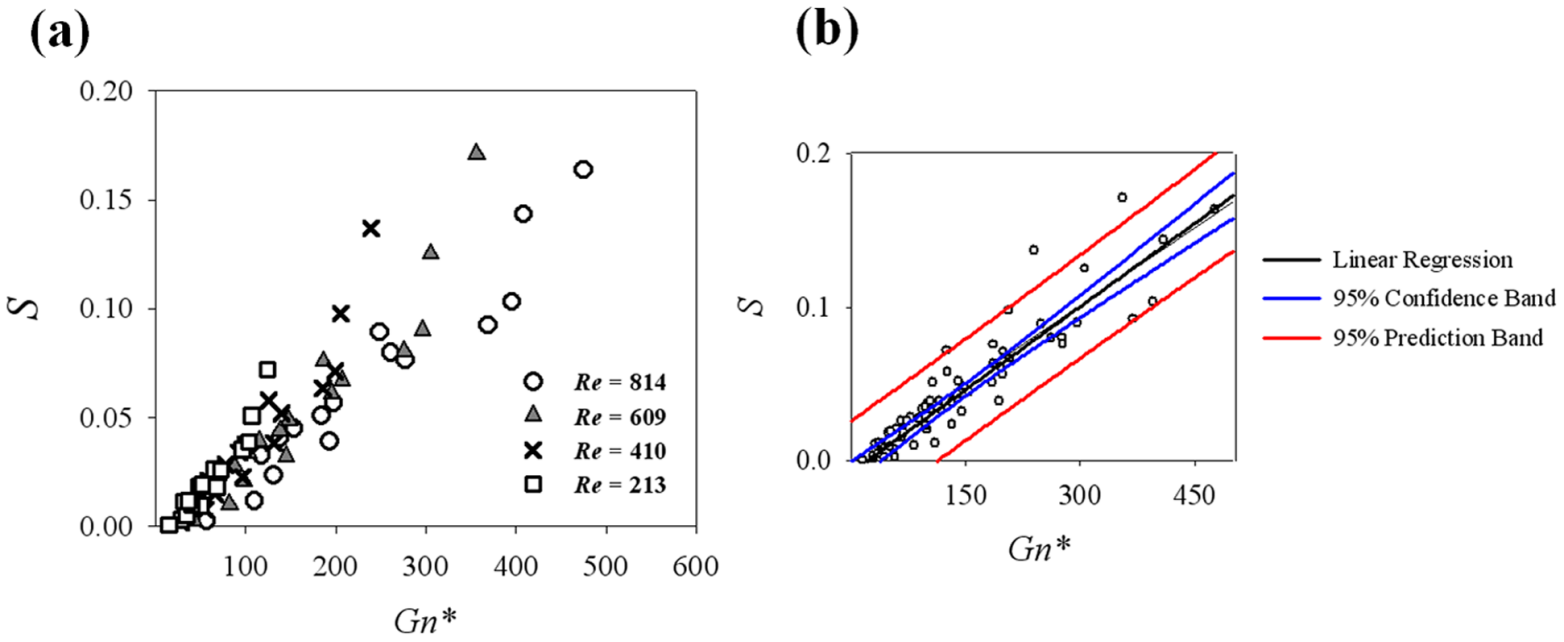
Variations of swirling intensity (*S*) with respect to Gn*. (a) Effect of *Re* on swirling intensity variation, (b) a linear regression curve (*S* = 0.0004×Gn*–0.0075, *R^2^* = 0.834) and 95% confidence and prediction bands.

**Figure 11:**
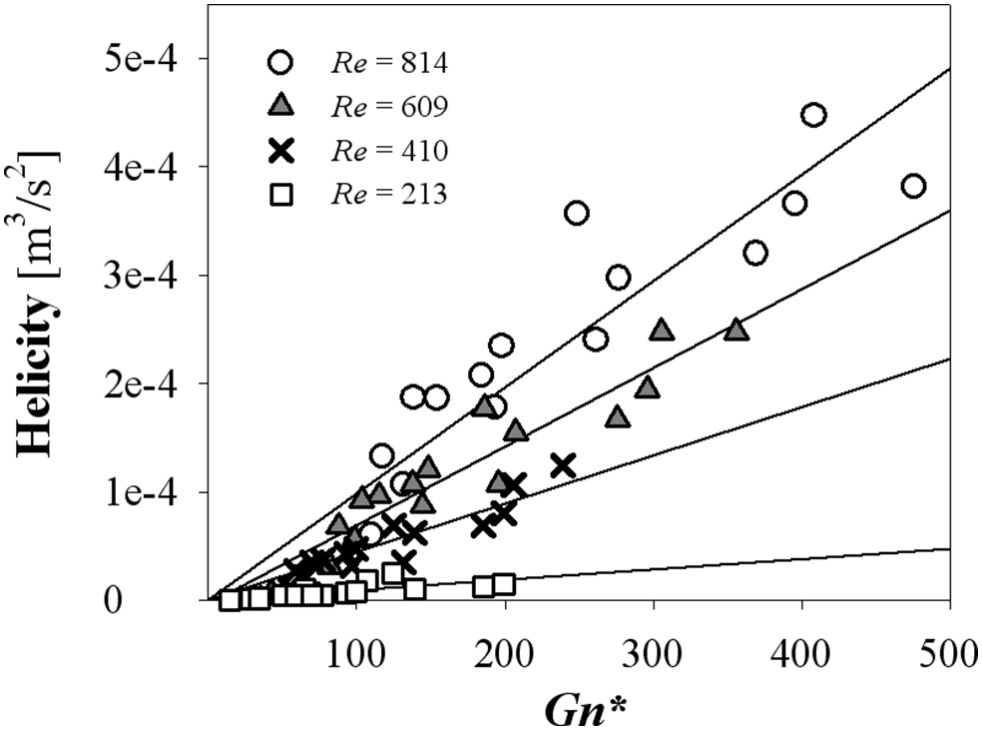
Helicity variations at the outlet surface with respect to Gn*.

The variations of swirling intensities obtained at all helical curvatures and pitches were analysed with the modified Germano number Gn* (figure 10). Regardless of *Re*, all *S* values are well overlapped and increase in proportion to Gn* (figure 10a). The linear regression line at 95% confidence and prediction bands (figure 10b) shows that *S* = 0.0004×Gn* − 0.0075 with *R^2^* = 0.834. Helicity, an alternative parameter of *S,* also shows a linear increment with Gn*. The slopes of the helicity increment are dependent on *Re* (figure 11). The slopes of the linear regression are 2.15×10^−7^ (*Re* = 213, *R^2^* = 0.859), 4.47×10^−7^ (*Re* = 410, *R^2^* = 0.879), 5.22×10^−7^ (*Re* = 609, *R^2^* = 0.896) and 9.82×10^−7^ (*Re* = 814, *R^2^* = 0.838).

**Figure 12:**
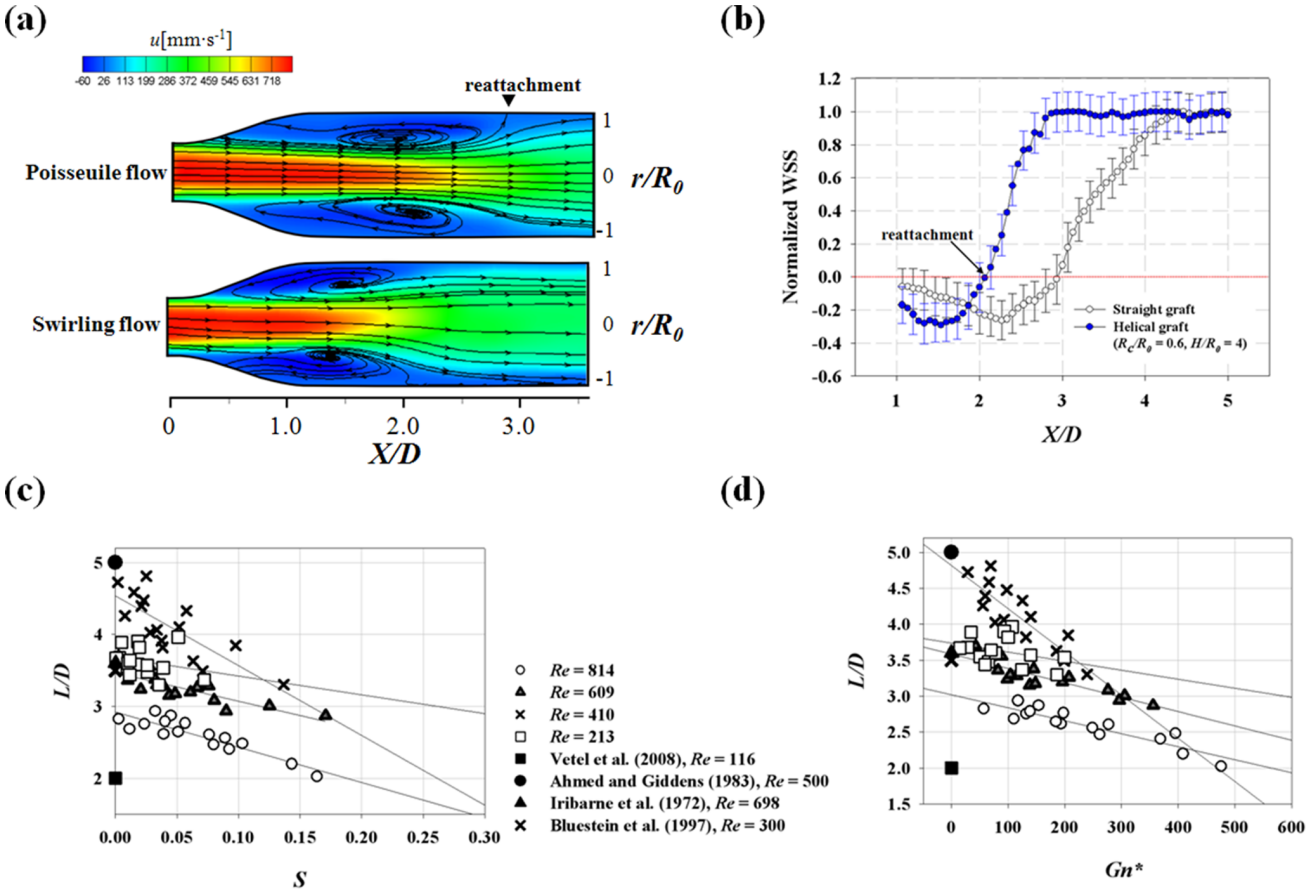
The effect of swirling flow on the length of flow reattachment (*L/D*) and WSS at the post-stenosis. (a) Velocity contours and streamlines at the post-stenosis. Poiseuille and swirling inlet flows are generated by the straight and helical tubes (*R_c_/R_0_* = 0.6, *H/R_0_* = 4) at *Re* = 814. (b) distribution of normalized WSS at the post-steonsis. WSS was normalized by the WSS that would exist in Poiseuille flow in a conduit at the same *Re.* The error bars indicate 95% confidence limits and only half of them are shown for clarity. (c) variations of *L/D* with respect to *S*, (d) variations of *L/D* with respect to Gn*. Mean standard deviations of *L*/*D* = 0.39.

The effect of the swirling flow in the helical tube on the post-stenosis flow was experimentally investigated using a PIV technique. The velocity contours and streamlines in figure 12a show that the swirling flow created by the helical tube (*R_c_/R_0_* = 0.6, *H/R_0_* = 4) significantly reduces the length of the jet flow and the recirculation area at the post-stenosis. As shown in figure 12b, the recirculation area is characterized by low negative WSS distribution, which is caused by the slow retrograde flow. While swirling flow has no significant influence on maximum and minimum magnitudes of the WSS, total area of the low WSS region is significantly reduced by the introduction of swirling flow due to reduction of the recirculation flow.

To consider the possible angular deviations in the reattachment lengths, eight reattachment lengths were obtained by measuring centreline flow fields at four different angles of the helical tube with an interval angle of 45°. The eight reattachment lengths (*L*) were averaged and normalized (*D*), and the relationship between the normalized reattachment (*L*/*D*) and *S* was analysed (figure 12c). The slopes of the regression lines are as follows: −2.62 (*R e* = 213, *R^2^* = 0.07), −9.71 (*Re* = 410, *R^2^* = 0.713), −3.97 (*Re* = 609, *R^2^* = 0.779) and −4.90 (*Re* = 814, *R^2^* = 0.816). At *Re*>213, *L/D* has a negative relationship with *S,* and the negative slope is the most dramatic at *Re* = 413. By contrast, the effect of *S* on *L/D* is statistically minor at *Re = *213. The magnitudes of *S* required to reduce *L/D* by half of *L/D* at *S* = 0 are as follows: 0.70 (*Re* = 213), 0.23 (*Re* = 410), 0.43 (*Re* = 713) and 0.30 (*Re* = 814). The same linear reduction of *L/D* with respect to Gn* was observed because of the positive relationship between *S* and Gn* (figure 12d). The slopes of the regression line are −1.24×10^−3^ (*Re* = 213, *R^2^* = 0.117), −5.99×10^−3^ (*Re* = 410, *R^2^* = 0.726), −1.99×10^−3^ (*Re* = 609, *R^2^* = 0.760) and −1.83×10^−3^ (*Re* = 814, *R^2^* = 0.833). The magnitudes of Gn* required to reduce *L/D* by half of *L/D* at Gn* = 0 are as follows: 1504.0 (*Re* = 213), 401.5 (*Re* = 410), 599.5 (*Re* = 713) and 834.3 (*Re* = 814). Meanwhile, the minimum *L/D* of the data is 2.02 at the maximum Gn* of 475.51 (*Re* = 814) with a helical tube of *R_c_/R_0_* = 0.6 and *H/R_0_* = 4. To compare *L/D* of the present data with the previous literature, the recirculation lengths obtained with non-swirling flows were shown in figure 12c-d. Direct comparison seems inadequate because the experimental set-up is slightly different. However, the *L/D* at small Gn* and *S* are comparably within the range of the previous literature [6], [29], [30], [40].

**Figure 13:**
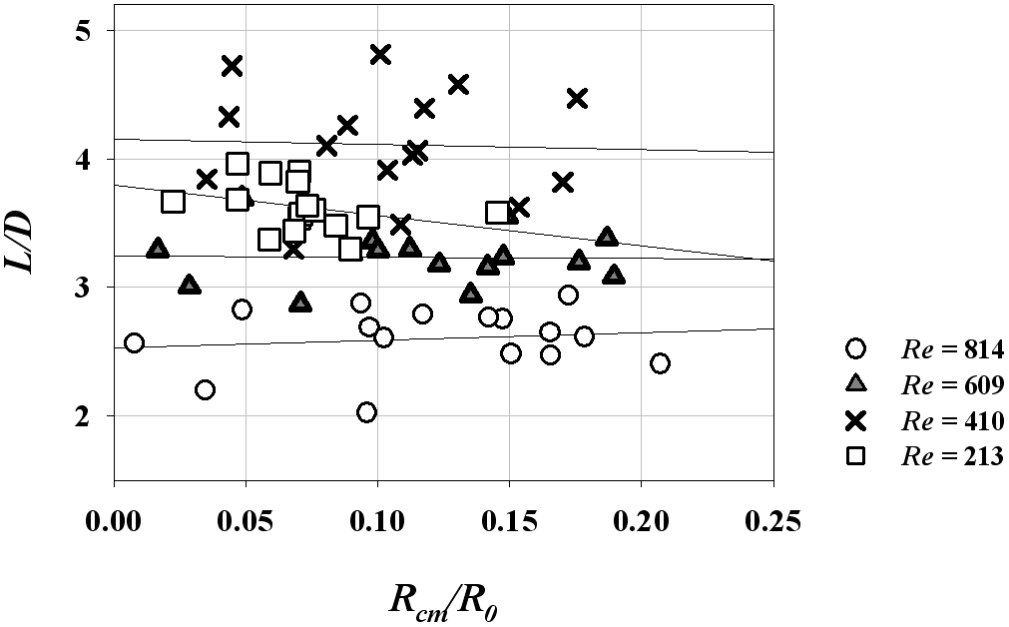
The effect of axial velocity skewness on reattachment length.

The effect of the axial velocity skewness on *L/D* was analysed to confirm whether the reduction of *L/D* was caused by the swirling secondary flow (figure 13). The skewness of the axial velocity profile was estimated according to the centre of the axial momentum flux as
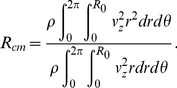
(14)


As a result of the torsion and curvature effect of the helical tubes, the normalized centre location of the axial momentum flux (*R_cm_/R_0_*) shifts up to 0.21 toward the wall. As shown in figure 13, axial velocity skewness has no significant effects on *L/D* reduction, and all regression lines have *R^2^*<0.1. This result indicates that the major contributor to *L/D* reduction is the swirling secondary flow and not the biased axial flow.

### 3.3 Effect of swirling flow on stenosis flow under a pulsatile flow condition

To confirm the effect of swirling flow on *L/D* reduction under the physiological pulsating flow condition, flow velocity fields at the post-stenosis were analysed under pulsatile flow (100 beats per minute) using a phase-locking PIV technique [41]. As inlet conditions for the stenosis, the swirling flow and Poiseuille flow were induced by a helical tube of *R_c_/R_0_* = 0.6 and *H/R_0_* = 4 and a straight tube, respectively. The pulsatile flow was generated by using a twin-pulsatile life support system (T-PLS, New heart bio.BHK, Korea).

**Figure 14:**
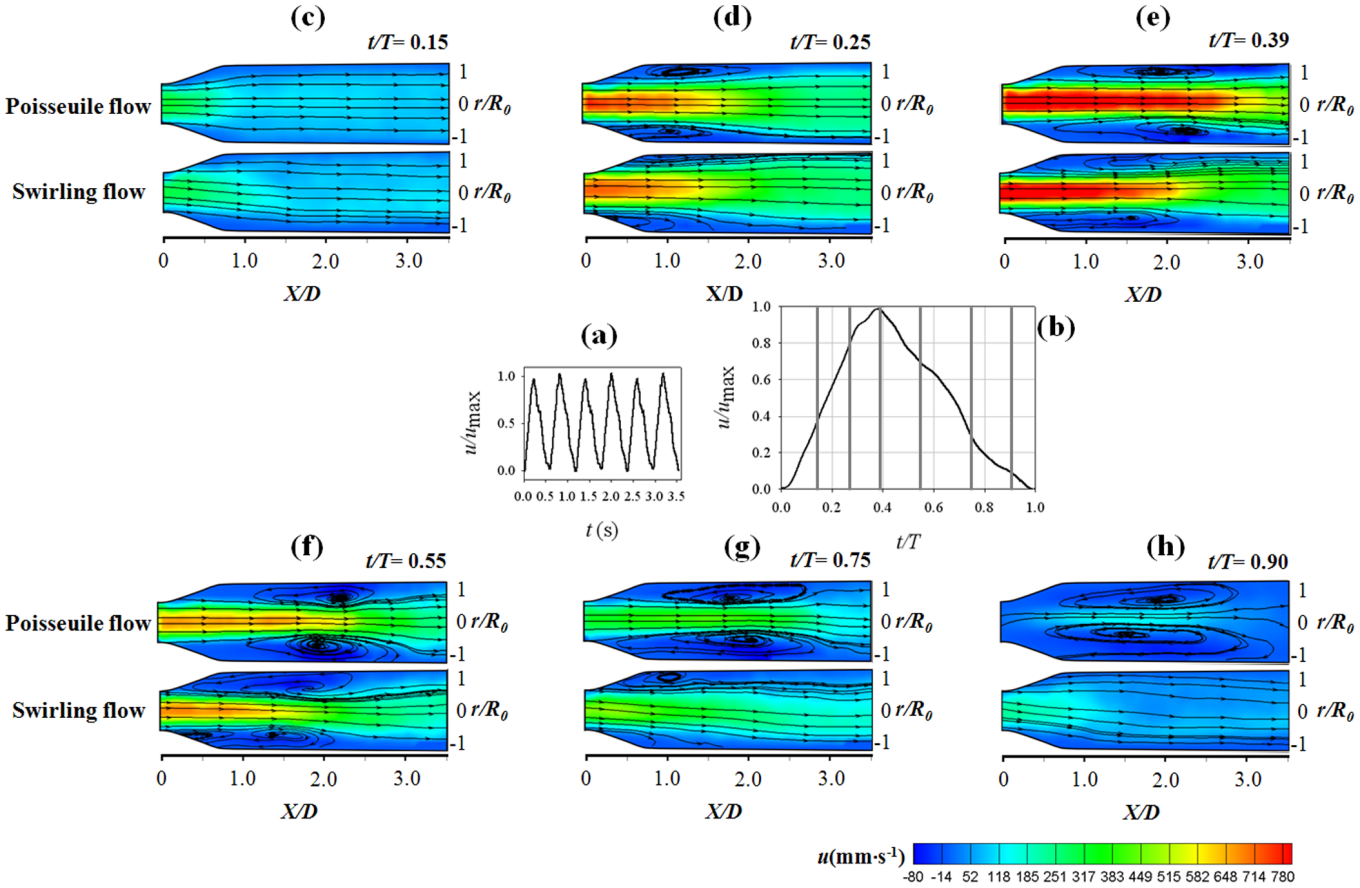
The effect of pulsatile swirling flow on flow structure at the post-stenosis. ( a) Pulsatile waveforms of the normalized velocity at the stenosis apex. The maximum *Re*, mean *Re* and Womersley number (α) of the flow are 860, 212 and 9.69, respectively. (b) Phase-averaged velocity waveform. Velocity contours and streamlines are shown at (c) *t/T* = 0.15, (d) *t/T* = 0.25, (e) *t/T* = 0.39, (f) *t/T* = 0.55, (g) *t/T* = 0.75, (h) *t/T* = 0.90. The Poiseuille flow (upper) and swirling flow (lower) are generated by the straight and helical tubes (*R_c_/R_0_* = 0.6, *H/R_0_* = 4).

Figure 14a shows the pulsatile waveforms of the normalized velocity at the stenosis apex. The maximum *Re*, mean *Re* and the Womersley number (*α*) of the flow are 860, 212, and 9.69, respectively. The phase-averaged velocity waveform obtained by ensemble averaging 55 cycles is shown in figure 14b. Among the pulsating cycles, the velocity contours and streamlines at six different phases (*t/T* = 0.15, 0.25, 0.39, 0.55, 0.75 and 0.90) are presented in figure 14c–h.

**Figure 15:**
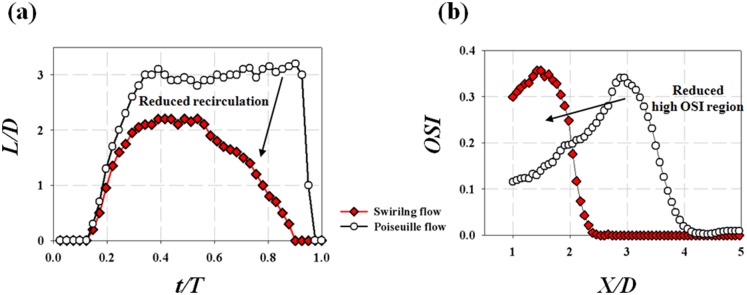
The effect of pulsatile swirling flow on the flow reattachment (*L/D*) and OSI at the post-stenosis. (a) Variations of flow reattachment (*L/D*) obtained from phase-averaged velocity fields, (b) OSI distribution at the post-stenosis. The Poiseuille flow (upper) and swirling flow (lower) are generated by the straight and helical tubes (*R_c_/R_0_* = 0.6, *H/R_0_* = 4).

At the early systole phase (*t/T* = 0.15), the jet flow starts to develop at the post-stenosis region. As the flow rate increases, the recirculation regions develop at *t/T* = 0.25. At *t/T* = 0.39, the length of the jet reaches the maximum at the peak flow rate. At this point, the swirling flow condition has a shorter jet flow length and a smaller recirculation flow area compared with the Poiseuille flow. At the diastole phase (0.39≤ *t/T*≤1.0), the recirculation flow at the swirling flow condition rapidly decreases along with the flow rate. On the contrary, the recirculation flow structure is maintained at *t/T = *0.90 when the Poiseuille flow serves as an inlet to the stenosis. Figure 15a quantitatively presents the variations of phase-averaged flow reattachment length (*L/D*). The result shows that both of the maximum and mean *L/D* are reduced up to 1.0D by the introduction of the swirling flow. This clearly shows that the swirling flow inhibits the development of the recirculation flow structure at the post-stenosis under the physiological pulsatile flow condition. Since the retrograde flow in the recirculation area induces relatively high WSS oscillation during the pulsating cycle, the size of the recirculation flow is directly related to the area of high OSI distribution at the post-stenosis. Figure 15b presents the effect of pulsatile swirling flow on the OSI distribution at the post-steonsis. The results shows that the introduction of the swirling flow confines the high OSI region to X/D<2.5 while the Poiseuille flow induces causes the high OSI region up to X/D<4.

### 3.4 Optimum design of helical graft for maximum swirling flow

As described in Sections 3.1 to 3.3, the swirling effect should be maximized to reduce *L/D* at the post-stenosis by designing a helical graft with the largest Gn* value. Therefore, the effective torsion β·η* of Gn* should be maximized by an optimum combination of *R_c_* and *H*. β·η* is defined as
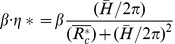
(15)


**Figure 16:**
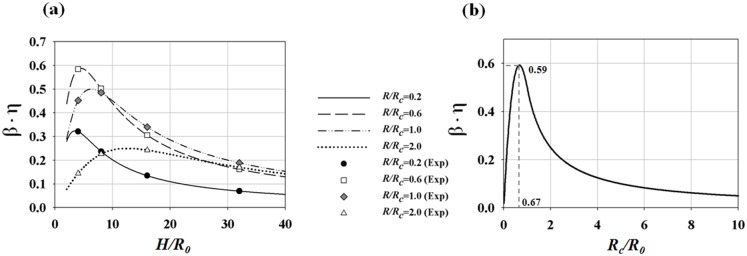
Variations of the effective torsion (β·η) curve for various helical curvatures and pitches. (a) Effect of helical pitch on *β·η*, (b) prediction of maximum *β·η* for a range of helical curvatures.



The β·η* curve for a constant *R_c_* has a local maximum point at a certain helical pitch, as shown in figure 16a. By differentiating β·η* with respect to the helical pitch,
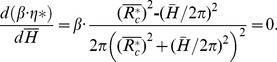
(16)
β·η* has a local maximum at 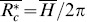. Then, the maximum β·η* at various *R_c_/R_0_* is obtained by

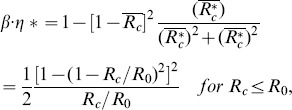
(17)

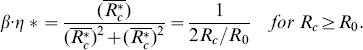
(18)


Following Eq. (17 to 18), the optimum design of the helical graft which provides the maximum swirling flow is predicted at *R_c_*/*R_0_* = 0.67 at *H*/*R_0_ = *4.72 (figure 16b). By contrast, when a helical pitch is pre-determined, an optimum *R_c_/R_0_* can be estimated by differentiating (15) with respect to *R_c_/R_0_*.

## Discussion

The present study investigated the swirling characteristics of the secondary flow produced by various shapes of helical tubes to reveal the potential advantages of a helical prosthetic vascular graft or a helical blood vessel. The influences of swirling flow on the flow field at the post-stenosis were experimentally measured by using a PIV technique. Specifically, the reduction on the recirculation length by the swirling flow was measured to estimate the beneficial effect of the swirling flow.

Vascular grafting is a widely performed surgical procedure wherein a prosthetic blood vessel is transplanted to bypass blood flow. Coronary arterial bypass grafting is performed to improve coronary circulation especially for high-risk patients with severe ventricular dysfunction, diabetes mellitus and so on [4]. Arteriovenous graft (AVG) is frequently performed to establish vascular access for patients undergoing hemodialysis. However, the use of vascular graft has significant complications which reduce long-term patency rate. Approximately 10% to 15% of vascular grafts occlude during the first year after operation, and approximately half of the grafts are only effective for a period of five to ten years [4], [42], [43]. The patency rate for AVG is also known to be lower than that for arteriovenous fistulas. The main cause of the failure is mostly attributed to the development of re-stenosis caused by IH at the graft–vessel junction [44].

Recently, helical-shaped vascular grafts have been found to generate little thrombosis and IH, whereas conventional grafts fail frequently because of the stenosis caused by IH. However, the fluid dynamic mechanism of the swirling flow in the blood vessel remains unclear. Coppola and Caro [13] reported that the combination of flow pulsatility and three-dimensionality generates the sweeping motion of the Dean vortices; this motion reduces the extremes of both the oxygen flux and the WSS on the vessel wall and ultimately serves as protection from diseases. Zhan *et al.* [26], [45] showed that platelet activation and adhesion are significantly suppressed under the swirling flow condition; their finding indicates the possible effect of swirling flow on thrombosis suppression.

While Caro et al. [9], [14], [46] have reported the ability of the swirling flow in the helical graft on suppressing the initial development of the stenosis, complete inhibitions of the IH development and thrombosis formation in the vascular graft could not be achieved to date. Therefore, the blood flow in the helical graft inevitably encounters local constriction of the blood vessel due to the development of IH, thrombosis formation or vasoconstriction [4], [21], [22]. At this point, the fluid-dynamic characteristics in the local constriction highly influence the further development of the stenosis or thrombosis [6], [47]–[49]. Therefore, in the present study, we considered that the helical graft is required to generate beneficial fluid-dynamic features at the post-stenosis region to suppress the progression of the vascular disease and decreases the risk of the graft failure.

The present study determined the fluid-dynamic performance of the helical graft by measuring reduction of the recirculation flow region at the post-stenosis. The recirculation flow is highly related to the progression of the disease and risk of the graft failure because of its pathological fluid-dynamic features, such as low WSS, high OSI, large residence time [33], [50]–[52]. The large particle residence time in the recirculation region is known to enhance platelet aggregation and thrombus formation at the post-stenosis region [6], [49], [53]. Ku *et al*. [47] reported that intimal thickening preferentially develops in regions with a very low magnitude of WSS and high OSI. Ishibashi *et al*. [54] also found that a strong correlation exists between the preferred site of intimal thickening in the anastomosed vessel and the slow recirculation flow region with low WSS. In addition, the probability for an atherosclerotic plaque at the apex of human vertebrobasilar junction was highly dependent on the size of the recirculation flow [48]. Therefore, the reduction amount of the recirculation flow is used as a reasonable index to indicate the fluid-dynamic performance of the helical graft at the stenotic environment.

The PIV velocity measurements illustrated in the stenosis model (figure 12) show that the swirling flow created by the helical graft significantly reduces the length of the recirculation flow. Since the recirculation area is characterized by low negative WSS distribution, reduction of the recirculation flow decreases total area of the low WSS region. In addition to the steady flow condition, the physiological pulsatile flow was also demonstrated in the study. The results reveal that unlike the Poiseuille flow condition, swirling flow significantly reduces the temporal development of the recirculation flow structure. In addition, high OSI region is also significantly decreased due to reduction of the recirculation flow (figure 15b). Therefore, this shows that pathological hemodynamic environments resulting from recirculation flow can be improved by the use of a helical graft and the improved hemodynamic characteristics may contribute to the increased patency rate of the helical graft.

To optimize the design of the helical graft, various helical configurations have been investigated. Van Canneyt *et al.* [55] numerically simulated the helical shape of five arteriovenous grafts with a pitch ranging from 35 mm to 105 mm and determined the performance of the helical graft by considering its ability to suppress disturbed shear distribution in the graft. Zheng *et al.* [56] also simulated the flows in helical grafts with various Dean numbers, helical pitches and amplitudes. They compared two helical grafts with the same Dean number and found that a short helical pitch and a large helical amplitude graft improve hemodynamic performance and induce a uniform WSS distribution on the graft wall. In addition, Cookson *et al.* [15] previously reported that fluid-mixing in the vascular graft is important for suppressing risk of thrombotic occlusion. They presented an optimum helical design by obtaining the best trade-off between fluid-mixing and pressure loss in the helical vascular graft. Recently, they carried out a computational study on fluid-dynamic characteristics in multiple helical geometries by using a coordinate transformation of the Navier-Stokes equations [57]. Their results show that combination of multiple helical geometries enhances fluid-mixing in the vascular graft with only small additional pressure losses.

The present study investigated various helical tubes with different helical curvatures and pitches and found that high-intensity swirling flow can be induced by an appropriate combination of a helical curvature and pitch, which results in the high magnitude of the helical parameter Gn*. As high Gn* can be obtained at the small radius of curvature (if the appropriate helical pitch is provided), the helical amplitude of the graft need not be large to achieve intense swirling flow. The theoretical prediction provided the maximum swirling flow by the helical tube at low helical curvature and short pitch (*R_c_/R_0_* = 0.67, *H/R_0_* = 4.72). In the experimental study, the maximum swirling flow was produced at the helical tube with *R_c_/R_0_* = 0.6 and *H/R_0_* = 4. This helical design closely matches the prediction in the experimental cases. The validity of the prediction is thus confirmed. Given that grafts with small curvature design are more robust and clinically applicable compared with those with large curvatures and short helical pitches, the optimum design suggested in this study would be advantageous for the future design of vascular grafts.

The performance of the helical graft in the present study was estimated from the fluid-dynamic velocity field information behind the local constriction. As the pathological mechanism of the graft failure is influenced by various biological and biomechanical factors such as abnormal function of ECs, overgrowth of smooth muscle cells and excessive platelet aggregation [4], the hemodynamic characteristics are one of the various pathological factors, thus the optimum design of the present study can be considered as a local optimum based on the fluid-dynamic point of view. This helical graft cannot be considered as a global optimum unless it satisfies multi-objective optimization. Therefore, clinical studies are required as a future work to demonstrate that the fluid-dynamically optimized helical graft is effective in suppressing the development of the IH and thrombosis and finally increasing the patency rate and longevity of the vascular graft.

Peterson and Plesniak [58] investigated the influence of inlet velocity profile and secondary flow on pulsatile flow in a model artery with stenosis. They compared flow structures at the post-stenosis under Poiseuille flow, skewed axial flow and skewed axial flow condition with the Dean-type secondary flow. Their study showed that curvature-induced secondary flow plays a minor role in the post-stenosis region compared with the skewed axial flow condition. However, the numerical simulation by Paul and Larman [32] predicted the significant effect of swirling flow on WSS distributions and turbulent dissipations at the stenosis. Ha and Lee [33] experimentally demonstrated that intense swirling flow significantly reduces the length of the recirculation flow region and enhances the early breakout of turbulent transition. The present study found that the swirling secondary flow has a strong influence on the recirculation flow at the post-stenosis. The study also determined that the skewing of the axial velocity profile has no statistically significant effects on recirculation length (figure 13).

Although this study mostly focused on flow characteristics in prosthetic tubes, the results can be expanded to characterize the flow in non-planar blood vessels and its effects on the circulatory system. Since the report on the existence of swirling flow, many researchers have attempted to use swirling flow as an indicator for estimating the development of CVDs. Houston *et al*. [59] compared two groups of patients with swirling flow and non-swirling flow in the abdominal aorta. They found that renal arterial stenosis is prevalent in patients without swirling flow. Mari *et al.* [60] proposed a method for quantifying the swirling type of secondary flow by using the colour Doppler imaging technique. Morbiducci *et al*. [11] also tried to quantify helical blood flow in vivo by using time-resolved cine phase-contrast magnetic resonance imaging (MRI). They suggested that the detection method for abnormalities in the development of helical flow structures can be used as a diagnostic/prognostic index in clinical practice. In the present study, we speculated that the occurrence of swirling flow and its intensity are mostly dependent on the helix-like 3D geometry of the blood vessel. Therefore, by inspecting the innate or acquired geometrical parameters of major blood vessels, patient-specific swirling flow can be estimated without complicated flow field measurements. Given that a modified Germano number Gn* can be easily calculated from the 3D geometrical parameters of blood vessels using advanced imaging techniques, such as X-ray computer tomography, ultrasound imaging and MRI, Gn* can be an effective index for detecting the tendencies of swirling flow and estimating the likelihood of disease development.

The present study has some limitations. The estimation of swirling flow using Gn* was based on the assumption of a helical tube with a circular cross-section. Therefore, swirling flows generated by a twisted pipe with a non-circular cross-section or a straight pipe with helical inserts cannot be interpreted with the Gn*. Although the present experimental model has a straight connection between the outlet of the helical tube and the stenosis channel, vascular grafts usually have complicated geometries, including the non-constant radius of curvatures and the large bifurcation angle at the junction with a host vessel. The influences of these geometries on the performance of helical grafts have yet to be established clearly. As the present study focused on the fluid dynamic characteristics of flow fields generated by helical grafts, the physiological performance of the helical graft suggested in the study, including its role in the development of thrombosis and IH, can be explored in the future.

## Conclusions

The present study investigated the swirling characteristics of the secondary flow produced by helical tubes with various shapes. The effects of the swirling flow on the flow field at the post-stenosis were investigated using a PIV velocity field measurement technique. Specifically, the reduction on the recirculation flow by the swirling flow was investigated to determine the beneficial effects of the graft. The intensity and helicity of the swirling flow were found to have a linear relation with the modified Germano number (Gn*) of the helical pipe. Furthermore, the high swirling flow was found to have a beneficial structure at the post-stenosis by reducing the size of the recirculation flow under steady and pulsatile flow conditions. The optimized helical design was predicted by estimating the magnitude of Gn*. The suggested optimal design is expected to benefit the design of vascular grafts.

## Supporting Information

Figure S1 **Schematic drawing of a helical pipe with a low radius of the curvature (** ***R_c_<R_0_*** **).** A correction factor (*β*) is introduced to take account of the reduced torsion effect and extend the use of *Gn* at *R_c_<R_0_*.
(DOCX)

Figure S2 **Schematic diagram of the cross-correlation PIV method.** A spatial cross-correlation between two consecutive flow images is used to determine the displacement and velocity of the flow.
(DOCX)
