# Supplementary material for: Fluid-Dynamic Optimal Design of Helical Vascular Graft for Stenotic Disturbed Flow
Source: PLoS One. 2014 Oct 31;9(10):e111047. doi: 10.1371/journal.pone.0111047 (PMC4215892; doi:10.1371/journal.pone.0111047)
Supplement: Figure S1 — Schematic drawing of a helical pipe with a low radius of the curvature ( Rc<R0 ). A correction factor (β) is introduced to take account of the reduced torsion effect and extend the use of Gn at Rc<R0. (DOCX) [file pone.0111047.s001.docx]

FIGURE S1. Schematic drawing of a helical pipe with a low radius of the curvature (*R_c_< R_0_)*


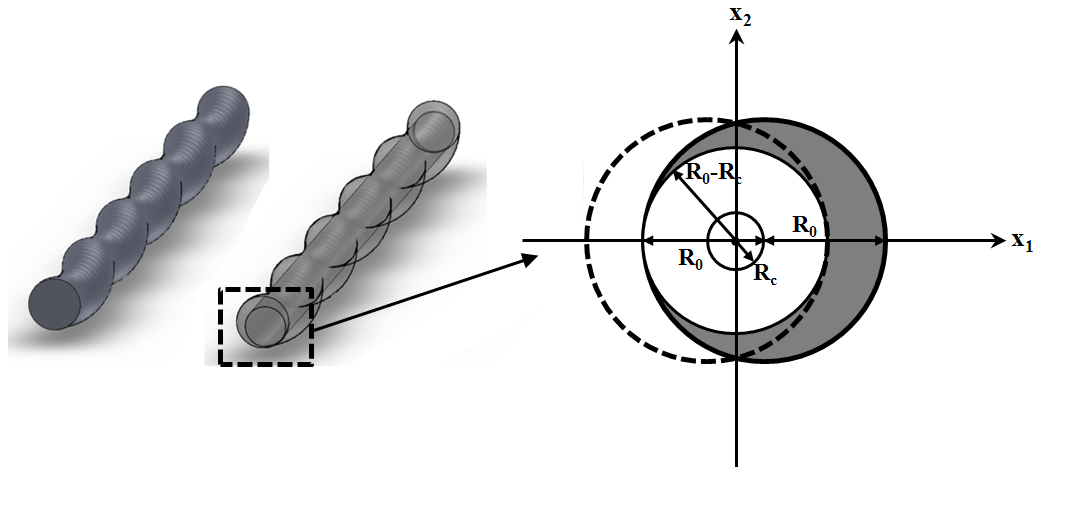


Since Germano derived *η* and *Gn* only for *R_c_> R_0_*, they need slight modification to be used for *R_c_< R_0_*. As shown in figure S1, the cross-section of the helical pipe at *R_c_< R_0_* can be considered to have helical-coiling area (grey) with non-coiling area (white). In the present study, it is assumed the helical-coiling area is an only contributor for generating the secondary flow in the helical pipe, therefore, the torsion effect of the helical pipe flow decreases when *R_c._* decreases and the non-coiling area increases. Therefore, a correction factor (*β*) is introduced to take account of the reduced torsion effect and extend the use of *Gn* at *R_c_< R_0_* as follows:

 (1)

In addition, the effective radius of curvature (*R_c_**) is also estimated only for the helical-coiling area by calculating the center of the helical area as follows:

*A_helical_·R_c_* + A_non-helical_·O = A·R_c_*  (2)

 (3)


 for *R_c_*≤ *R_0_* (4)

 for *R_c_*≥ *R_0_* (5)
