# Supplementary material for: Fluid-Dynamic Optimal Design of Helical Vascular Graft for Stenotic Disturbed Flow
Source: PLoS One. 2014 Oct 31;9(10):e111047. doi: 10.1371/journal.pone.0111047 (PMC4215892; doi:10.1371/journal.pone.0111047)
Supplement: Figure S2 — Schematic diagram of the cross-correlation PIV method. A spatial cross-correlation between two consecutive flow images is used to determine the displacement and velocity of the flow. (DOCX) [file pone.0111047.s002.docx]

**FIGURE S2. Schematic diagram of the cross-correlation PIV method**

**
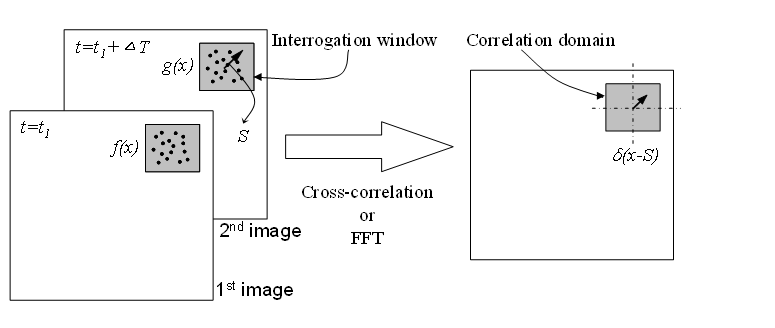
**

A PIV velocity field measurement technique estimates velocity vectors from a pair of particle images. Fig.S1 shows the schematic diagram of the cross-correlation PIV method. A spatial cross-correlation between two consecutive flow images is used to determine the displacement and velocity of the flow [[1](#_ENREF_1)]. Assuming that the first image sample (within the interrogation window) of two successive flow images has a spatial distribution expressed as a brightness function, and the flow moved an displacement of *S* in a short time interval (Δ*t*) between the two image frames, then the brightness distribution of the sampled region in the next image is given as *g(x) = f(x-S)*. By calculating the two-dimensional Fourier transforms of the two regions, the displacement *S* can be evaluated. Given *f(x)* and *g(x)*,

$\begin{matrix} F\{f(x)\}=\int_{x} f(x)\exp[-j2\pi(k\cdot x)]dx\text{ } \\ F\{g(x)\}=F\{f(x-S)\}=F\{f(x)\}\exp[j2\pi(k\cdot S)]\text{ } \end{matrix}$ (S1)

where, *k* is the spatial frequency or wave number. Displacement *S* can be determined from the following relation:

$F\left\{\frac{F\{f(x)\}}{F\{g(x)\}}\right\}=F\{\exp[j2\pi(k\cdot S)]\}=\int_{x} \exp[-j2\pi k\cdot(x-S)]dk=\delta(x-S)\text{ }$ (S2)

where, *δ(x – S)* is the Dirac delta function. This is a complex division of each corresponding pair of Fourier coefficients. This new set of coefficients is then again transformed to obtain the Dirac delta function which represents the displacement vector *S*. Subsequently the displacement *S* is divided by the time interval (Δ*t*) to extract the velocity information of the interrogation window.

The PIV velocity data for uncertainty analysis were obtained in the horizontal plane. The average velocity field was obtained by averaging 450 instantaneous PIV instantaneous vector fields. The centerline velocity and flow rate are measured to 247.3 ± 1*.*5 mm s^−1^. Over the majority of the velocity profile, the error in the centerline velocity is less than ±1%. The error increases to approximately ±5% near the channel wall. In the nearest wall region, the relative error becomes larger (~ 40%) because of the velocity gradient within the interrogation window.

Reference

1. Adrian RJ , Westerweel J (2011) Particle Image Velocimetry. Cambridge ; New York: Cambridge University Press. xxvi, 558 pages p.
